# Supplementary material for: Reinstating motivational states: Electrical signatures of craving and neural mind reading
Source: PLoS One. 2026 Jan 2;21(1):e0315068. doi: 10.1371/journal.pone.0315068 (PMC12758794; doi:10.1371/journal.pone.0315068)
Supplement: Attachment — Submitted filename: Rebuttal letter.docx [file pone.0315068.s002.docx]

PONE-D-24-53416

Decoding Motivational States and Craving through Electrical Markers for Neural 'Mind Reading’

PLOS ONE

Dear Editor.

We are sincerely grateful for the opportunity to revise our manuscript. We have carefully addressed each point raised by Reviewer 1 and Reviewer 2 and have revised the manuscript accordingly. We are confident that the substantial revisions, both theoretical and statistical, have greatly enhanced the quality of the manuscript. Thanks for the time devoted to carefully consider our manuscript.

We have corrected two figures, carried out all the requested statistical analyses, revised some of the localizations, fine-tuned the discussion, and thoroughly revised both the introduction and the discussion sections, reducing the emphasis on localization. Most importantly, we have added the missing information concerning the experimental paradigm, the timing of the mental and physiological simulation for each category, the way in which the states to be recalled were concatenated, as well as all the technical parameters of the LORETA analysis and all other requested details.

In addition, we have clarified and elaborated on the feasibility of the imaginative paradigm, which appears to be state-of-the-art, rigorous, and reliable from multiple perspectives. We hope that the manuscript is now substantially improved and meets the expectations of both the editor and the reviewers.

**Editorial comments**

*1) “ATE – Fondo di Ateneo No. 31159-2019-ATE-0064, University of Milano-Bicocca”*

**AUTHOR:** The funders had no role in study design, data collection and analysis, decision to publish, or preparation of the manuscript.

This information was added to the revised manuscript.

*2. In the online submission form, you indicated that “All data generated or analyzed during this study are included in this published article and its supplementary information files. The data supporting the findings of this study are available from the corresponding author* [*mado.proverbio@unimib.it*](mailto:mado.proverbio@unimib.it) *upon request. Due to privacy restrictions, some data may not be shared publicly.”*

**AUTHOR**: The revised manuscript now explicitly states that the stimulus set and related materials are accessible through this open-access repository.

All data generated or analyzed during this study are included in this published article and its supplementary information files. The stimulus set and experimental procedure, provided to ensure replicability, are available in the open repository: Proverbio, Alice Mado; Zanetti, Alice (2025). An auditory-mediated communication paradigm for evaluating individual needs and motivational states in locked-in patients. Bicocca Open Archive Research Data, V1. <https://doi.org/10.17632/f2mg2n6cwv.1>

**Reviewer #1:**

Major Suggestions for Minor Revision:

*Graphical Clarity in Figures 4 and 7
While the data visualizations are generally effective, I recommend improving the readability of the axis labels in Figures 4 and 7, particularly along the x-axis. These appear overly compressed or poorly contrasted, especially when compared to the more legible formatting seen in Figure 9. For consistency and accessibility, the same graphical standards should be uniformly applied across all figures.*

**AUTHOR**: Thank you for this helpful comment. We have revised Figures 4 and 7 to improve graphical clarity, ensuring that the axis labels—especially along the x-axis—are now more readable and visually consistent with the formatting used in Figure 9.

*Effect Size Reporting in ANOVAs
To enhance the statistical transparency and reproducibility of the findings, I strongly encourage the authors to report effect size metrics—specifically partial eta squared (η²) and epsilon (ε) values—for all ANOVAs performed. While significance testing provides valuable information, effect sizes offer a more nuanced understanding of the strength and practical relevance of observed differences. Given the complexity and richness of the ERP data across multiple states and components, effect size indices are essential for readers to assess the robustness and scope of the effects reported.*

**AUTHOR:** Thank you for this valuable suggestion. We have now included effect size metrics—specifically partial eta squared (η_p_^2^) and epsilon (ε) values—for all ANOVAs reported in the manuscript, thereby enhancing the statistical transparency and reproducibility of our findings.

*Subsectioning the Discussion
The Discussion would greatly benefit from clearer structure through the use of sub-paragraphs or thematic subheadings. Given the breadth of the results—ranging from component-specific findings (e.g., N400, P400) to source localization and state-by-state interpretations—organizing the discussion around these axes would improve clarity and reader engagement. Suggested subheadings might include: Component-Specific Effects, State-Specific Activations, Theoretical Implications, and Future Directions.*

**AUTHOR:** Thank you for this insightful recommendation. We have reorganized the Discussion section by introducing thematic subheadings that structure the content around key interpretative axes, including component-specific effects, state-specific activations, theoretical implications, and future directions. This restructuring improves the clarity and readability of the section.

*Minor Language and Style Adjustments
The manuscript is generally well-written and the scientific language is appropriate. However, I suggest minor improvements to fluency and idiomatic expression. For instance:

Page 10, line 287:

Instead of: "Has it been mentioned (in this experimental sequence) to desire a nice plate of pasta with meat sauce?" I suggest: "Has the notion of desiring a plate of pasta with meat sauce emerged at any juncture during the course of this experimental sequence?" This revised phrasing better aligns with the formal tone of the manuscript and enhances clarity.*

**AUTHOR:**

The sentence has now been revised for clarity and accuracy.

Has anyone mentioned wanting a nice plate of pasta with meat sauce?

**Reviewer #2**: Unfortunately, despite its strong sides, the study suffers from some conceptual and methodological weaknesses, which, in my opinion, prevent publication. Most important are pointed out below:

*1. Aim is presented as detecting markers of different motivational and physiological states. However, the study uses imagery and does not induce these states. However, while the relationship between imagery and real states is covered in the introduction, the described aim of the study remains misleading.*

**AUTHOR.** We appreciate the reviewer’s comment and the opportunity to clarify this point. As correctly noted, the present study employed a **mental imagery paradigm** to elicit representations of distinct motivational and physiological states. This approach was deliberately chosen to ensure both **ethical feasibility** and **experimental control**, given that directly inducing strong visceral or affective states (e.g., severe hunger, pain, or sadness) would not be acceptable in a laboratory setting.

Importantly, as extensively discussed in the Introduction, **imagery of bodily or emotional states engages neural networks that largely overlap with those activated during actual experience**, as shown in previous neuroimaging and electrophysiological studies (e.g., Suess and Abdel Rahman, 2015; Mustile et al., 2024). Therefore, the use of vivid state imagery provides a valid and well-established proxy for accessing the corresponding motivational and physiological representations.

Mustile M, Kourtis D, Edwards MG, Donaldson DI, Ietswaart M. Neural correlates of motor imagery and execution in real-world dynamic behavior: evidence for similarities and differences. Front Hum Neurosci. 2024 Jun 21;18:1412307. doi: 10.3389/fnhum.2024.1412307.

Suess F, Abdel Rahman R. Mental imagery of emotions: Electrophysiological evidence. Neuroimage. 2015 Jul 1;114:147-57. doi: 10.1016/j.neuroimage.2015.03.063. Epub 2015 Apr 1. PMID: 25842292.

It would be both ethically unacceptable and methodologically impracticable to directly induce states such as severe starving, heat or pain in healthy participants. Indeed, no such experimental studies exist in the literature, precisely because deliberately eliciting visceral discomfort or physical suffering would raise serious ethical concerns. Conversely, there is a vast body of research conducted on clinical or addicted populations, where genuine, non-simulated craving states can be observed without experimental induction (e.g. craving for drugs, cocaine, tobacco or food).

In our study, the goal was to capture the imagined simulation of such motivational and physiological states, as vividly and semantically as possible, in order to explore whether it is feasible to identify and classify their corresponding neural signatures. This approach allows the investigation of the cerebral representation of internal states while fully respecting ethical constraints and participants’ well-being.

To address the reviewer’s concern we have revised the study’s aim as follows:

<<The aim of the present electroencephalographic (EEG) study was to identify neural markers associated with the **mental representation of distinct motivational and physiological states**, including visceral needs, affective and somatosensory experiences, and secondary goals. Rather than directly inducing such states — which would be both ethically unacceptable and methodologically unfeasible — we employed a **guided mental imagery paradigm** in which participants vividly recalled or simulated specific conditions (e.g., thirst, hunger, pain, joy) in response to **auditory-verbal cues** accompanied by evocative soundscapes congruent with each state. This approach allowed us to investigate whether **distinct neural signatures** could be detected and classified for each internally represented state, thereby advancing the possibility of developing **EEG-based markers for affective and motivational decoding**, with potential applications in brain–computer interface (BCI) systems for non-communicative patients.>>

2. *The introduction seems to only partially cover the current literature, and sometimes the findings seem to be picked up from the literature pool to support a particular claim rather than present the existing views. For example, citing data showing that activation of mPFC is related to positive scenarios, while many studies support the role of this area in self-referential processes with no regard to valence.*

**AUTHOR:** The introduction has been thoroughly revised. A clearer distinction has now been made between the frontopolar and ristro/medial prefrontal areas and the medial prefrontal cortex, and previous inaccuracies have been corrected. Thank you very much for raising this important point.

The table 2 has been revised to ensure greater precision. Indeed, by considering the X, Y, Z Talairach coordinates of the activations, a more accurate localization within the same Brodmann area can be achieved.

In line with the reviewer’s suggestion, we have reduced the emphasis on the description of the functional properties of the areas identified as active by LORETA, considering the relatively limited spatial resolution of swLORETA.

This was achieved by modifying the Discussion and adding a statement in the Limitations section.

Katayama N, Nakagawa A, Umeda S, Terasawa Y, Kurata C, Tabuchi H, Kikuchi T, Mimura M. Frontopolar cortex activation associated with pessimistic future-thinking in adults with major depressive disorder. Neuroimage Clin. 2019;23:101877. doi: 10.1016/j.nicl.2019.101877.

*I am also concerned about the number of different states that the Authors wanted to induce during the relatively long procedure. I doubt whether it was possible to keep participants really involved and deeply following such diverse and altering imagery states. Especially since these states are expected to induce specific bodily states.*

We fully understand the reviewer’s skepticism—indeed, we would share such concerns ourselves if the study had not allowed sufficient time for the mental evocation of each state, or if it had required participants to shift motivational representations abruptly within a few seconds. We have now clarified in the revised manuscript that each condition included a **dedicated imagery period of approximately five seconds** preceding the sustained phase of representation, and that **each motivational state remained stable for a minimum of 20–26 seconds**. This clarification highlights a key methodological strength and a distinctive feature of our paradigm.

We did not expect macroscopic bodily changes to occur, but rather **mental representations** of such states. However, the reviewer is correct in noting the temporal aspect of state induction. In fact, the sequences were designed so that each representation would **persist for at least 15–20 seconds**.

Since each stimulus plus ISI lasted 5200 ms, four repetitions of the same motivational state lasted about 21 seconds, and five repetitions approximately 26 seconds. This important methodological detail has now been added to the revised manuscript. We thank the reviewer for bringing this point to our attention.

Below is an example of a typical sequence (sequence 12).

Additionally, we randomized the order of the stimuli to ensure that **opposite or semantically related states** (for example, hunger and thirst, joy and sadness) never appeared in close temporal proximity.

Run12

cold14

cold15

cold16

cold17

cold34

fear3

fear27

fear28

fear6

movem.19

movem.11

movem.13

movem.23

heat19

heat20

heat1

heat3

joy14

joy25

joy15

joy16

sleep28

sleep29

sleep30

sleep8

sleep10

music8

music32

music33

music34

thirst1

thirst2

thirst5

thirst6

Thanks to the reviewer’s insightful comment, this crucial methodological detail—previously omitted for brevity—has now been explicitly included in the revised version of the manuscript.

<<The auditory sequences were designed to ensure both the vivid evocation and the temporal stability of each motivational or physiological representation. Each stimulus plus inter-stimulus interval (ISI) lasted 5200 ms. The same motivational state was presented in **four or five consecutive repetitions**, resulting in a total duration of approximately **20–26 seconds** for each state. This temporal design allowed participants sufficient time to form and maintain a vivid mental representation before the next condition began. In addition, to prevent semantic interference or emotional carryover, **the order of presentation was fully randomized** with the constraint that **opposite or conceptually related states** (e.g., hunger, thirst, sleepiness) were never presented in close temporal proximity.>>

3. *The components of the evoked potentials are interpreted as responses for imagery. This is also seriously problematic for me, as the exact timing and intensity for imagery cannot be determined. I’m afraid that the discussed ERP components are the responses for a cue (frame) as the only well temporally defined stimulus.*

**AUTHOR**: This is the typical imagery paradigm, featuring a command or inducer and a cue to synchronize ERP recording. The imagery can start slightly before or after the cue, which serves purely for synchronization purposes. Most imagery studies adopt this paradigm — it is standard practice and represents the current state of the art (see also: Proverbio, 2024).

In fact, precedent ERP work has **reliably isolated imagery-related components** using this same cue-then-imagery architecture across **visual, auditory, motor, and emotional imagery**. Classic studies (e.g., Farah et al., 1989; Wu et al., 2011) time-locked ERPs to imagery generation and show modality-appropriate effects (e.g., imagery LPC tracking imagined pitch/loudness; visual-imagery effects on face identification at later matching stages). More recent work on visual and auditory imagery of biological relevant stimuli, such as faces, bodies, animals (e.g., Proverbio et al., 2023) explicitly **prompts imagery with a light signal** to synchronize imagery onset for ERP averaging.

Collectively, these data show that it is both methodologically standard and empirically feasible to observe **imagery-evoked ERPs** that are **not** reducible to the initial cue/frame. Our design follows these best practices and our results match the **component topographies, latencies, and content-specific modulations**, reported in that literature. We bet to disagree with the reviewer on this specific point

Farah MJ, Weisberg LL, Monheit M, Peronnet F. Brain Activity Underlying Mental Imagery: Event-related Potentials During Mental Image Generation. J Cogn Neurosci. 1989 Fall;1(4):302-16. doi: 10.1162/jocn.1989.1.4.302.

Wu J, Yu Z, Mai X, Wei J, Luo Y. Pitch and loudness information encoded in auditory imagery as revealed by event-related potentials. Psychophysiology. 2011 Mar;48(3):415-9. doi: 10.1111/j.1469-8986.2010.01070.x.

Proverbio AM, Tacchini M, Jiang K. What do you have in mind? ERP markers of visual and auditory imagery. Brain Cogn. 2023 Mar;166:105954. doi: 10.1016/j.bandc.2023.105954.

Proverbio AM. The temporal dynamics of visual imagery and BCI: Comment on "Visual mental imagery: Evidence for a heterarchical neural architecture" by Spagna et al. Phys Life Rev. 2024 Mar;48:174-175. doi: 10.1016/j.plrev.2024.01.006.

4. *The Authors do not convince me that they observe imagery-evoked responses.*

The evidence clearly indicates imagery-evoked activity; cue-related explanations are untenable given the identical visual input across conditions.

Since a yellow frame is displayed on the screen for **all motivational states**, any divergence or difference between the ERP waveforms that is **not random**—that is, any effect that changes **systematically as a function of category** according to rigorous statistical analysis—must necessarily be attributed to the participant’s **imagery-related activity**, to their **mental state**. I trust that the reviewer will agree with this reasoning.

5. *The parameters and settings of source localization are not reported at all. This is not trivial, as localization methodology is very susceptible to improper settings.*

**AUTHOR**: thanks for raising this point. This information is now provided to the reader

Both the head model's segmentation and generation were executed using Advanced Neuro Technology, a software program developed by ASA (Zanow and Knösche, 2004). The swLORETA analysis yielded results indicating the statistical activation of a subset comprising 1056 dipoles. These results provide information on both their Magnitude values (in nA) and spatial coordinates following the Talairach & Tournoux (1988) system. A dipole, in this context, represents the potential difference between the basal and apical portions of a cell, resulting in a positive and negative difference with an orthogonal orientation to the magnetic field. Utilizing the Collins dipole table each electromagnetic dipole was associated with its respective Gyrus and Brodmann Area. Source space properties were: grey matter; grid spacing = 5 mm; estimated SNR= 3; Leadfield Normalization: weighted

Zanow, F., and Knösche, T. R. (2004). ASA–Advanced Source Analysis of continuous and event-related EEG/MEG signals. Brain Topogr. 16, 287–290.

6. *The final analysis includes only 23 participants, which is a small sample and does not keep up with the current standards. Especially facing the number of analyses (variables and their levels).*

We fully acknowledge the reviewer’s concern regarding the relatively limited sample size. This is indeed a valid point, and we have therefore re-examined all statistical analyses to strengthen the robustness of our results. Specifically, we have now applied the **Greenhouse–Geisser correction** (reporting the corresponding ε values) to account for potential violations of sphericity in repeated-measures designs.

In addition, we have computed and reported **partial eta squared (partial η²)** as a measure of effect size for each main effect and interaction. The partial η² quantifies the proportion of total variance in the dependent variable that is attributable to a specific factor, **after controlling for the variance explained by other factors in the model**. In other words, partial η² expresses the magnitude of an effect independent of the sample size, providing a more informative index of the practical significance of the findings.

Together, the application of the Greenhouse–Geisser correction and the inclusion of partial η² values make the statistical conclusions more rigorous, transparent, and reliable, thereby mitigating concerns related to the relatively modest sample size (N = 23).

7. *It would be beneficial to report not only anova differences between categories but also confusion matrices, as the aim of the study was focused towards mind reading.*

Indeed, we conducted a dedicated machine learning investigation (on a fewer number of electrode to avoid overfitting) specifically targeting this issue, which has been just **successfully published!**

Colafiglio, T., Lombardi, A., Di Noia, T., De Bonis, M. L. N., Narducci, F., **Proverbio, A. M.** (2025). Machine learning classification of motivational states: Insights from EEG analysis of perception and imagery. Expert Systems with Applications, 275, 127076. <https://doi.org/10.1016/j.eswa.2025.127076>

F1 scores resulting from the classifiers for the imagery condition with 18 channels configuration

In the imagery condition using a 14-channel configuration, the highest accuracy was observed for the pair ‘cold/fear’ with an accuracy of 0.82. The pair ‘happiness/hunger’ also maintains a high accuracy of 0.80. The overall accuracy for other imagined states was more variable, with some pairs such as ‘thirst/pain’ and ‘music/hot’ showing moderate accuracies of 0.65 and 0.47, respectively. This variability highlights the challenge of vividly imagining certain states compared to perceiving them.

This approach — in which the imagery-related ERP waveforms were classified using three machine-learning models (Support Vector Machine, Random Forest, and a feedforward Neural Network) — is, however, primarily engineering-oriented, as it focuses on the AI automatic recognition of the activated mental category, a goal that was achieved with appreciable heuristic success.
